# Prandial–basal insulin regimens plus oral antihyperglycaemic agents to improve mealtime glycaemia: initiate and progressively advance insulin therapy in type 2 diabetes

**DOI:** 10.1111/j.1463-1326.2010.01287.x

**Published:** 2010-11

**Authors:** S M Jain, X Mao, M Escalante-Pulido, N Vorokhobina, I Lopez, L L Ilag

**Affiliations:** 1 TOTALL Diabetes Hormone InstituteIndore, India; 2 Eli Lilly and Company, Lilly Corporate CenterIndianapolis, IN, USA; 3 Unidad Medica de Alta Especialidad (UMAE), Centro Medico Nacional de Occident (CMNO), Instituto Mexicano del Seguro Social (IMSS), Department of EndocrinolgyGuadalajara, Mexico; 4 State Institution of Professional Education, St Petersburg Medical Academy of Postgraduate Studies under the Federal Agency of Health Care and Social DevelopmentSt Petersburg, Russian Federation; 5 Medical Practice of Ignacio LopezMantes La Jolie, France

**Keywords:** basal and bolus therapy, insulin glargine, insulin lispro, prandial premixed therapy, type 2 diabetes mellitus

## Abstract

**Aims:** To compare two progressive approaches [once-daily insulin glargine plus ≤3 mealtime lispro (G+L) vs. insulin lispro mix 50/50 (LM50/50) progression once up to thrice daily (premix progression, PP)] of beginning and advancing insulin in patients with type 2 diabetes (T2D) and inadequate glycaemic control on oral therapy, with the aim of showing non-inferiority of PP to G+L.

**Methods:** Patients were randomized to PP (n = 242) or G+L (n = 242) in a 36-week, multinational, open-label trial. Dinnertime insulin LM 50/50 could be replaced with insulin lispro mix 75/25 if needed for fasting glycaemic control.

**Results:** Baseline haemoglobin A1c (HbA1c) were 9.5% (PP) and 9.3% (G+L); p = 0.095. Change in A1C (baseline to endpoint) was −1.76% (PP) and −1.93% (G+L) (p = 0.097) [between-group difference of 0.17 (95% confidence interval: −0.03, 0.37)]. Non-inferiority of PP to G+L was not shown based on the prespecified non-inferiority margin of 0.3%. A1C was lower with G+L at weeks 12 (7.8 vs. 7.9%; p = 0.042), 24 (7.4 vs. 7.6%; p = 0.046), but not at week 36 (7.5 vs. 7.6%; p = 0.405). There were no significant differences in percentages of patients achieving A1C ≤7%, overall hypoglycaemia incidence and rate or weight change. Total daily insulin dosages at endpoint were higher with PP vs. G+L (0.57 vs. 0.51 U/kg; p = 0.017), likely due to more injections (1.98 vs. 1.79; p = 0.011).

**Conclusions:** Both treatments progressively improved glycaemic control in patients with T2D on oral therapy, although non-inferiority of PP to G+L was not shown. Higher insulin doses were observed with PP with no between-treatment differences in overall hypoglycaemia or weight gain.

## Introduction

Intensive glycaemic control can delay the onset and slow the progression of diabetes-related complications in patients with type 2 diabetes (T2D) [1,2]. The recently published 10-year follow-up of the United Kingdom Prospective Diabetes Study showed that early intervention with intensive glucose control had a ‘legacy effect': early intensive glucose control continued to reduce microvascular complications, and also reduced the risk for myocardial infarction and all-cause mortality during 10 years of post-trial follow-up [3].

Recent studies support the clinical utility of initiating insulin therapy early in patients with T2D by adding a single injection of basal insulin to an existing oral regimen in order to achieve and maintain target hemoglobin A1C (HbA1c) levels ≤7% [4–8]. As only 28–58% of patients starting on basal insulin analogues in these studies achieved A1C levels <7% [8], ≤7% [5–7] or ≤7.5% [4], there is a potential opportunity to improve blood glucose (BG) levels by adding a rapid-acting insulin at mealtime as recommended if basal insulin analogue is insufficient [9]. The 3-year results of the Treating to Target in Type 2 Diabetes (4-T) study suggest that most patients are likely to need a second type of insulin [10].

Premixed formulations that contain both basal and prandial insulin could also be used in starting or progressing insulin therapy [5,11] but need more study [9,12]. Of patients receiving twice-daily injections of premix (i.e. biphasic) insulin, ∼42–48% achieved A1C <7% [8] or ≤7% [5,13], suggesting potential room for improvement if the regimen is intensified. In subjects without diabetes, ∼50% of daily insulin secretion is basal and the remainder is postprandial [14]. Thus, an insulin lispro mix of 50% insulin lispro protamine suspension and 50% insulin lispro (LM50/50) more closely reflects physiologic insulin secretion than other premixed formulations and is hypothesized to provide reasonably similar clinical outcomes as a separately dosed basal with bolus insulin regimen.

In this study, two progressive approaches to starting and intensifying insulin therapy are compared in patients with T2D and inadequate glycaemic control on oral therapy: premix insulin progression (PP) with once- then twice- or thrice-daily LM50/50 insulin administration vs. basal insulin glargine initiated once daily then supplemented with one to three prandial insulin (lispro) injections [insulin glargine plus insulin lispro (G+L)] as needed to meet glycaemic targets.

## Research Design and Methods

This 36-week, randomized, open-label, active-controlled trial was conducted in nine countries (Australia, Canada, France, Greece, India, Republic of Korea, Mexico, Russian Federation and Spain) in accordance with the provisions of the Declaration of Helsinki and Good Clinical Practice guidelines. All patients provided written informed consent. Eligible patients were men and women, 30–80 years, with T2D, A1C 7.5–12.0% using ≥2 oral antihyperglycaemic medications (OAMs) for ≥90 days, insulin naïve, capable and willing to use insulin injection devices and self-monitoring of blood glucose (SMBG) levels. Patients were excluded if they had ≥1 episode of severe hypoglycaemia within the prior 6 months, body mass index (BMI) >40 kg/m^2^, were taking a thiazolidinedione dose greater than what was indicated in combination with insulin, or were taking glucose-lowering agents other than metformin, sulphonylurea or thiazolidinedione, had functional capacity class III/IV cardiac disease, impaired renal function, active liver disease, or serum alanine transaminase levels >2 times the upper limit of normal.

### Study Medications and Treatments

**Figure 1:**
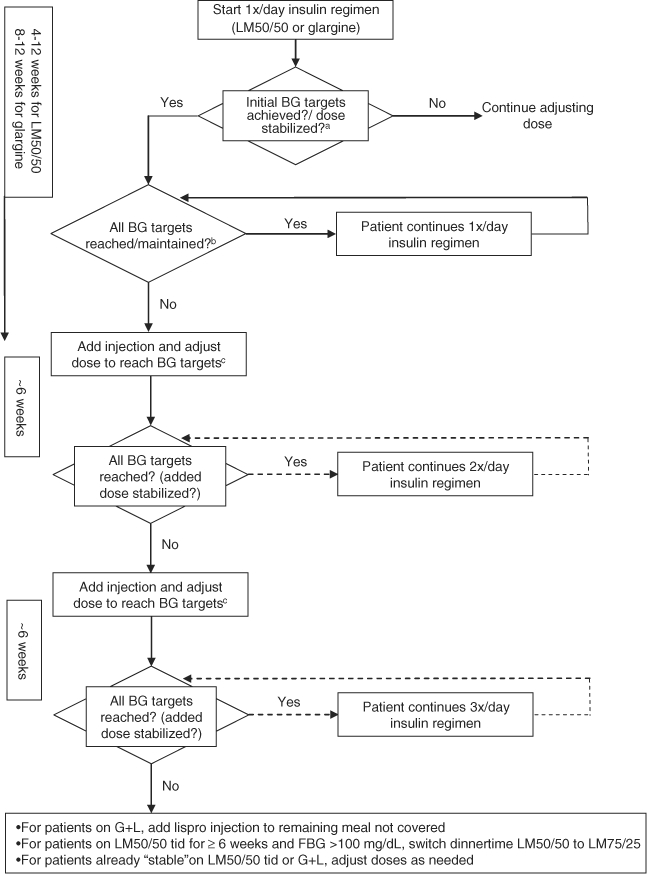
Insulin intensification flow chart. ^a^At start, adjust insulin glargine based on fasting blood glucose (FBG); adjust LM50/50 based on bedtime blood glucose (BG). ^b^Premeal BG [4.4–5.6 mmol/l (80–100 mg/dl)] and bedtime BG [4.5–6.1 mmol/l (81–110 mg/dl)]. ^c^Based on premeal and bedtime BG readings, the appropriate insulin injection was added at the meal preceding the episode of hyperglycaemia. For example, for a patient with elevated BG before dinner, an insulin injection at lunchtime would be introduced (dose based on BG reading and corresponding dose recommended in Table 1). G+L, insulin glargine plus lispro; LM50/50, insulin lispro mix 50/50; LM75/25, insulin lispro mix 75/25.

Patients were randomized to treatment by country and stratified within country by sulphonylurea use and A1C (≤8.5 and >8.5%) through an interactive telephone system. Most patients started on either one 10 U injection of LM50/50 administered with the locally available insulin pen, mostly HumaPen® Luxura™ pen (Eli Lilly and Company, Indianapolis, IN, USA) within 15 min prior to the evening meal or one 10 U injection of insulin glargine (Lantus®) administered with the locally available insulin pen, mostly OptiPen® Pro 1 or Lantus® OptiSet® (Sanofi-Aventis, Paris, France) at approximately the same time each morning. In either group, 12 U was the starting dose if fasting blood glucose (FBG) was ≥10 mmol (180 mg/dl). Insulin dose adjustments were made utilizing regimen-specific insulin dose titration algorithms (Tables 1 and 2) to achieve target FBG and preprandial BG levels <5.5 mmol/l (<100 mg/dl). Reasonable periods of time were permitted for regimen titration and stabilization (Tables 1 and 2; figure 1). No additional injections were allowed after week 24 to allow A1C stabilization with a particular regimen at study end (36 weeks); insulin dose adjustments based on existing injections were allowed. Patients continued their prestudy OAM regimens during the study.

**Table 2:** Insulin dose titration algorithm for insulin glargine.*

FBG from preceding 2 days†		Dose change‡
(mg/dl)	(mmol/l)	(U)
<80	<4.4	−2
81–100	4.5–5.5	0
101–120	5.6–6.6	+2
121–140	6.7–7.7	+4
141–160	7.8–8.8	+6
≥161	≥8.9	+8

FBG, fasting blood glucose in plasma-equivalent value.

* The insulin glargine dose should not be increased if hypoglycaemia occurred during the previous week.

† Based upon the average of at least two readings.

‡ Each patient's dose should be assessed by the investigator at least on a weekly basis and adjusted as needed for the first 10 weeks of the study. Thereafter, dose adjustments may occur at least once every 2 weeks for the next 8 weeks, then every 3 weeks for the remaining 18 weeks of the study. Total insulin dose should not be increased by more than 10 U/day or 10% of the total daily insulin dose, whichever is greater.

**Table 1:** Insulin dose titration algorithm for LM50/50 or insulin lispro.

Bedtime BG (mg/dl)*		Fasting and preprandial BG (mg/dl)*		Starting next day prandial dose change†
(mg/dl)	(mmol/l)	(mg/dl)	(mmol/l)	(U)
<80	<4.4	<80	<4.4	−2
81–110	4.5–6.1	81–100	4.5–5.5	No change
111–139	6.2–7.7	101–139	5.6–7.7	+2
140–179	7.8–9.9	140–179	7.8–9.9	+4
≥180	>9.9	≥180	>9.9	+6

BG, blood glucose in plasma-equivalent value; LM50/50, insulin lispro mix 50/50.

* Investigators may request additional BG monitoring from patients and assess other glucose values at other times when making dose-adjustment decisions.

† Each patient's dose should be assessed by the investigator at least on a weekly basis and adjusted as needed for the first 10 weeks of the study. Thereafter, dose adjustments may occur at least once every 2 weeks for the next 8 weeks, then every 3 weeks for the remaining 18 weeks of the study. Total insulin dose should not be increased by more than 10 U/day or 10% of the total daily insulin dose, whichever is greater. The prandial dose change is applied to the meal immediately preceding the BG being targeted. For example, the LM50/50 or insulin lispro dose at breakfast is adjusted based on the prelunch BG; the lunchtime insulin dose based on the predinner BG and the dinnertime insulin dose based on the bedtime and/or fasting BG reading.

Hypoglycaemia events were assessed as to incidence (proportion of patients who experience hypoglycaemia), rate (per person per 30 days) and severity. Hypoglycaemia was defined as any time a patient experienced an associated sign or symptom, or had a BG level of <3.9 mmol/l (<70 mg/dl), even if it was not associated with signs, symptoms or treatment. Nocturnal hypoglycaemia was defined as any hypoglycaemic event that occurred between bedtime and waking. Severe hypoglycaemia was defined as an episode with symptoms consistent with hypoglycaemia in which the patient required the assistance of another person, and was associated with either a BG level of <2.8 mmol/l (50 mg/dl) or prompt recovery after oral carbohydrate, glucagon or intravenous glucose.

### Outcome Measures

The primary efficacy measure was change in A1C from baseline to endpoint. Secondary efficacy measures were A1C, percentages of patients achieving A1C ≤6.5, <7 and ≤7.0% and 7-point SMBG profiles over time; insulin dose (total, basal and prandial); number of injections per day; and safety, including hypoglycaemia (described earlier), weight change and treatment-emergent adverse events (TEAEs). Blood, urine and serum samples were collected at screening (week 2). A1C was analysed by a central laboratory (Covance, Princeton, NJ, USA).

### Statistical Methods

The sample size for the primary analysis was calculated on the basis of a two-sided test for non-inferiority with a 5% significance level. Assuming a standard deviation of 1.1% for A1C, 213 patients completing the study per treatment group would provide 80% power to meet the prespecified non-inferiority limit of 0.3%. Patients who completed a 36-week A1C measurement constituted the per-protocol analysis population (primary analysis).

The primary outcome was analysed by an analysis of covariance (ancova) model with treatment, country, baseline A1C, baseline A1C stratum and sulphonylurea use as covariates. If the upper limit was below 0.3% (PP−G+L), then PP was non-inferior to G+L. Secondary outcomes (A1C at other time points, SMBG, glycaemic variability, total insulin dose and number of injections) were analysed by ancova model, on the intent-to-treat (ITT) dataset, with treatment, country, baseline A1C stratum, sulphonylurea use and baseline as covariates. The percentages of patients achieving A1C goals (≤7.0, <7.0 and ≤6.5%) were analysed by logistic regression analysis with terms for treatment, country, sulphonylurea use and baseline A1C.

Safety assessments were based on the entire randomly assigned population. The proportion of patients reporting at least one hypoglycaemic event or a severe hypoglycaemic event was compared using Fisher's exact test. Hypoglycaemic rate and severe hypoglycaemic rate were analysed using ranked analysis of variance (anova) with treatment, country, baseline A1C stratum and sulphonylurea use as covariates. Categorical safety variables were compared between groups with Fisher's exact test.

## Results

### Patient Disposition and Baseline Characteristics

**Figure 2:**
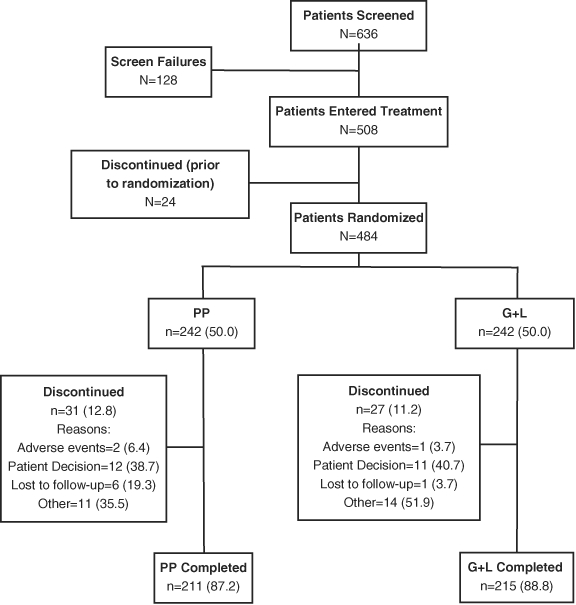
Patient disposition, the number (%) of patients. Other reasons for discontinuation were entry criteria not met, protocol violation, physician decision, death and sponsor decision. G+L, insulin glargine plus insulin lispro; PP, premix progression (insulin lispro mix 50/50).

Patient disposition is shown in figure 2. A total of 211 (87.2%) patients in the PP group and 215 (88.8%) patients in the G+L group completed the study. Patient demographic and baseline characteristics were similar between the PP and G+L groups for all measures except FBG, which was significantly lower in the G+L vs. PP group (Table 3).

**Table 3:** Baseline demographics and characteristics of randomly assigned patients.

	Treatment group	
	+ L (n = 195)	PP (n = 188)
Age (years)	59.9 ± 9.6	58.9 ± 8.8
Sex (male : female)	101 (51.8) : 94 (48.2)	86 (45.7) : 102 (54.3)
Race/ethnicity		
Caucasian	116 (59.5)	110 (58.5)
Hispanic	32 (16.4)	33 (17.6)
Black/African descent	0 (0.0)	1 (0.5)
East Asian	20 (10.3)	19 (10.1)
West Asian	27 (13.8)	25 (13.3)
(Indian subcontinent)		
Weight (kg)	78.8 ± 15.2	78.2 ± 15.3
BMI (kg/m^2^)	28.8 ± 4.5	29.1 ± 4.4
Diabetes duration (years)	12.0 ± 7.3	11.4 ± 5.6
A1C (%)	9.3 ± 1.2	9.5 ± 1.2
FBG† (LSM ± s.e.)		
mmol/l	9.6 ± 0.9	10.2 ± 0.9*
mg/day/l	172.8 ± 16.2	183.6 ± 16.2*
Concomitant OAMs at study entry		
Met/Sulph/TZD	21 (10.8)	20 (10.6)
Met/Sulph	163 (83.6)	163 (86.7)
Met/TZD	2 (1.0)	0 (0.0)
Sulph/TZD	9 (4.6)	5 (2.7)

Data are given as means ± s.d. or n (%) unless otherwise indicated.

A1C, hemoglobin A1C; BMI, body mass index; FBG, fasting blood glucose; G+L, insulin glargine plus insulin lispro; LSM, least-squares mean; Met, metformin; n, the number of patients; OAM, oral antihyperglycaemic agent; PP, premix progression (insulin lispro mix 50/50); s.d., standard deviation; s.e., standard error; Sulph, sulphonylurea; TZD, thiazolidinedione.

* p = 0.014; all other comparisons were not significantly different.

† Calculated from the intent-to-treat population; all other values in the table are based on the per-protocol population.

### Glycaemic Control

**Figure 3:**
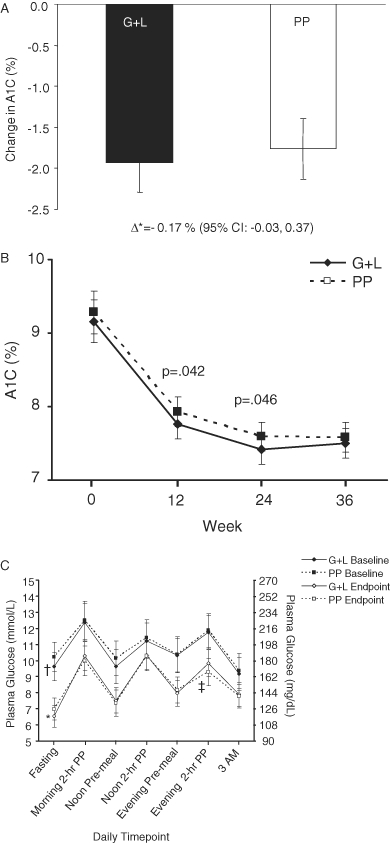
(A) Change in mean A1C ± s.e.m. from baseline to endpoint for G+L and PP groups and the difference (G+L − PP) in A1C change with the 95% confidence interval (CI). (B) Mean A1C ± s.e.m. over the study for G+L and PP groups. (C) SMBG 7-point profiles at baseline and endpoint for patients treated with G+L or PP; ^*^p = 0.014 for comparison of baseline fasting values between treatment groups; †p = 0.010 for comparison of endpoint fasting values between treatment groups; ‡p = 0.010 for comparison of endpoint evening 2-h PP values between treatment groups. A1C, hemoglobin A1C; G+L, insulin glargine plus lispro; PP, premix progression (insulin lispro mix 50/50); s.e.m., standard error of mean; SMBG, self-monitored blood glucose.

Baseline A1C was similar in both groups (Table 3). Non-inferiority of PP to G+L was not achieved for A1C change from baseline to endpoint as the upper limit of the 95% confidence interval (CI) (−0.03, 0.37) was >0.3% (figure 3A). The A1C change from baseline to endpoint [least-squares mean (LSM) ± standard error (s.e.)] was −1.76 ± 0.37% for the PP group (n = 188) and −1.93 ± 0.36% for the G+L group (n = 195), a between-group difference that was not statistically significant (0.17%; p = 0.097). Over the course of the study, A1C (LSM ± s.e.) declined in both groups (figure 3B). At weeks 12 (PP, 7.93 ± 0.20%; G+L, 7.76 ± 0.20%; p = 0.042) and 24 (PP, 7.59 ± 0.20%; G+L, 7.42 ± 0.20%; p = 0.046), A1C was lower in the G+L vs. PP group; however, by week 36, A1C values were not statistically different between groups (PP, 7.58 ± 0.20%; G+L, 7.50 ± 0.20%; p = 0.405). There were no significant differences between groups at endpoint in the percentages of patients achieving A1C ≤7% (PP, 36.8%; G+L, 43.0%; p = 0.227), <7% (PP, 35.0%; G+L, 39.1%; p = 0.482) and ≤6.5% (PP, 13.2%; G+L, 19.1%; p = 0.108), nor at any 12-week interval (data not shown).

At baseline, SMBG values (LSM ± s.e.) were similar between groups at all time points except fasting, which was significantly lower in the G+L vs. PP group [9.6 ± 0.9 mmol/l (172.8 ± 16.2 mg/dl) vs. 10.2 ± 0.9 mmol/l (183.6 ± 16.2 mg/dl); p = 0.014] (figure 3C). At endpoint, both therapies significantly reduced 7-point SMBG values from baseline; fasting values were significantly lower in the G+L vs. PP group [6.5 ± 0.7 mmol/l (117.0 ± 12.6 mg/dl) vs. 7.0 ± 0.7 mmol/l (126.0 ± 12.6 mg/dl); p = 0.010], and evening postprandial values were significantly lower in the PP vs. G+L group [9.8 ± 0.8 mmol/l (176.4 ± 14.4 mg/dl) vs. 9.3 ± 0.9 mmol/l (167.4 ± 16.2 mg/dl); p = 0.010].

Among patients in the PP group, at endpoint (n = 234): 75 (32.1%) patients were on LM50/50 once daily (A1C: 7.52), 65 (27.8%) patients were on LM50/50 twice daily (morning + evening; A1C: 7.51), 17 (7.3%) patients were on LM50/50 twice daily (mid-day + evening; A1C: 7.28), 66 (28.2%) patients were on LM50/50 thrice daily (A1C: 7.61) and 11 (4.7%) patients were on LM50/50 (morning) + LM50/50 (mid-day) + LM75/25 (75% insulin lispro protamine suspension/25% insulin lispro) (evening) (A1C: 7.27). Among patients in the G+L group at endpoint (n = 235): 108 (46.0%) patients were on insulin glargine once daily (A1C: 7.25), 62 (26.4%) patients were on G+L once daily (A1C: 7.24), 48 (20.4%) patients were on G+L twice daily (A1C: 7.69) and 17 (7.2%) patients were on G+L thrice daily (A1C: 6.85). Thus, only patients treated with G+L thrice daily achieved a mean A1C <7.0%; only a small number of patients were advanced to this regimen.

### Insulin Dose, Number of Injections and Weight Gain

Weight-adjusted total daily insulin dosages (LSM ± s.e.) at endpoint were significantly greater for the PP vs. G+L group (0.57 ± 0.11 vs. 0.51 ± 0.11 U/kg; p = 0.017). Most patients ended up taking one or two injections (PP, 67%; G+L, 72%; p = 0.229). Mean number (LSM ± s.e.) of insulin injections at endpoint was significantly greater in the PP vs. G+L group (1.98 ± 0.3 vs. 1.79 ± 0.29; p = 0.011). Body weight (LSM ± s.e.) change from baseline at endpoint was similar in both groups (PP, 3.09 ± 1.44 kg; G+L, 3.19 ± 1.42 kg; p = 0.803).

### Safety: Hypoglycaemia and Adverse Events

The incidence of overall (over the treatment period) all hypoglycaemia [PP, 74.5% (n = 178); G+L, 74.6% (n = 179); p = 1.00], nocturnal hypoglycaemia [PP, 46.9% (n = 112); G+L, 46.7% (n = 112); p = 1.00] and severe hypoglycaemia [PP, 3.4% (n = 8); G+L, 2.1% (n = 5); p = 0.416] was similar in both groups. The incidence of all hypoglycaemia at endpoint [last observation carried forward (LOCF)] broken down by number of injections is provided in Table 4; statistical comparisons were not made because of low numbers.

**Table 4:** Incidence of hypoglycaemia at endpoint (LOCF) by number of injections.

		Number of injections				
		1	2	3	4	5*
G+L	n	110	65	48	16	1
	Hypoglycaemia, n (%)	44 (40)	31 (48)	22 (46)	9 (56)	0 (0)
PP	n	65	94	80	NA	NA
	Hypoglycaemia, n (%)	26 (40)	30 (32)	31 (39)	NA	NA

The incidence of hypoglycaemia was defined as the number of patients with at least one hypoglycaemic episode.

G+L, insulin glargine plus lispro; LOCF, last observation carried forward; n, the number of patients; NA, not applicable; PP, premix progression (insulin lispro mix 50/50).

* One patient received two injections of insulin glargine (one dose in the morning and one in the evening) plus three lispro injections.

There was no statistically significant difference between the two groups for the overall all or overall nocturnal hypoglycaemic rate. Higher rates (mean ± s.d.) of hypoglycaemic episodes were observed with patients in the G+L group at LOCF endpoint (2.19 ± 3.60 vs. 1.57 ± 2.98 episodes per patient per 30 days; p = 0.022), corresponding to hypoglycaemia occurring between the 24th and 36th week of treatment.

Overall, 88 (36.4%) patients in the PP group and 92 (38.0%) patients in the G+L group experienced at least one TEAE during the study (p = 0.778). A small percentage of patients in each group experienced a serious adverse event during the study: 11 (4.5%) patients in the PP group and 10 (4.1%) patients in the G+L group (p = 1.00). There were three deaths reported in the study (PP, n = 0; G+L, n = 3); none were considered to be related to study drug or device [coronary artery disease (n = 1), pulmonary edema (n = 1) and undetermined (n = 1)].

## Discussion

More evidence for the use of specific insulin regimens in T2D is needed beyond the introduction of the starting insulin regimen. This study evaluated two progressive regimens for advancing insulin therapy in insulin-naïve patients with T2D continuing prestudy OAMs. Both regimens started with once-daily insulin injections and provided increasing mealtime insulin coverage with additional injections, and both resulted in clinically relevant decreases in A1C over the course of the study. While there was no statistically significant difference between groups in A1C reduction at endpoint and non-inferiority was not shown, important observations in each approach may be noteworthy to consider during clinical management.

During the first 24 weeks, while patients in the G+L group were injecting once-daily insulin glargine and adding another injection, patients' mean A1C was significantly lowered in the G+L vs. PP group. From the 24th to 36th week, when additional mealtime insulin injections were not introduced but dose titrations were allowed, A1C increased slightly in the G+L group and, by endpoint, there were no significant differences between the groups. Most studies evaluating basal insulin added to OAMs in T2D note a decrease in A1C from baseline up to ∼12 weeks, after which they stabilize [4,5]; whereas, in the present study, the improvement in A1C continued in the G+L group until 24 weeks. Thus, additional efficacy in lowering BG was observed while additional mealtime insulin was introduced. The G+L regimen more effectively lowered FBG compared to the PP regimen, which is consistent with other studies comparing premix to basal insulin analogues [5,8,11,12], and prandial premixed therapy to basal/bolus therapy [15].

In the PP group, A1C decreased from baseline to 24 weeks almost in parallel with the G+L group, although levels were slightly higher. While PP patients had higher A1C at the 12th and 24th week, patients in this group were able to maintain their A1C levels between the 24th and 36th week; by the end of week 36, there was no difference in A1C between groups. The PP regimen more effectively lowered evening postprandial values compared to G+L therapy. A greater number of patients in the PP vs. G+L group were receiving more than one injection by the end of week 24, which may explain why A1C levels were maintained in this group.

The higher rate of hypoglycaemia in the G+L vs. PP group occurred after week 24, a point after which no additional mealtime lispro injections could be introduced. Because A1C increased slightly in the G+L group between weeks 24 and 36, the increased rate of hypoglycaemia is unlikely to be the result of lower glucose levels but may have resulted from increasing the insulin dose without adding injections. In contrast, the greater number of injections in the PP group may have allowed administration of higher doses overall without increasing hypoglycaemia. This finding is consistent with the rationale behind multiple daily insulin (MDI) injections (i.e. four injections per day with separate basal and bolus components), where each injection provides a unique opportunity for more flexible dose adjustment [16] to improve glycaemic control while avoiding hypoglycaemia.

Our findings are consistent with those of Rosenstock et al. [15] in which LM50/50 with each meal and separately dosed basal/bolus therapy both significantly lowered A1C and was also unable to show non-inferiority. Some important differences and similarities between the two studies are noteworthy. The Rosenstock et al. study included patients already taking insulin and randomized to fixed and intensive regimens with three to four insulin injections, whereas the insulin-naïve patients in our study were started on one injection then gradually escalated to up to three to four injections until week 24. While most patients advanced therapy to more than one injection, not many reached three to four injections; yet within 24–36 weeks, mean A1Cs had dropped to ∼7.5%. Considering that patients in the Rosenstock et al. study had mean A1C of ∼9.0% at baseline while on once-daily basal insulin (∼50 units/day), our results highlight the potential improvement attainable by advancing to more than one injection or providing mealtime insulin coverage to optimize glycaemic control. While greater weight gain has been reported with premixed vs. basal insulin regimens [12], there were no weight differences between regimens in the Rosenstock et al. [15] study or the present study. There were also no differences in hypoglycaemia between regimens in either study.

An important difference between the two studies is the mean A1C levels at study endpoint. With the intensive insulin therapies in the Rosenstock et al. study, mean A1C at endpoint was <7.0% with both treatments; whereas in the present study, mean A1C at endpoint was 7.6% while patients were on a median of two insulin injections. The difference in achieved mean A1Cs at endpoint again highlights the potential for improvement in glycaemic control by advancing prandial insulin to cover all meals.

Relatively few other studies have evaluated progressive insulin advancement in the context of a clinical trial. The Orals Plus Apidra and LANTUS (OPAL) study [17] evaluated treatment intensification in German patients with T2D not optimally controlled on insulin glargine plus OAMs by adding a single injection of rapid-acting insulin analogue glulisine before breakfast or the main mealtime. This approach also led to significant reductions in A1C. One observational study [18] employed a progressive titration of biphasic insulin aspart (NovoLog® Mix 70/30; Novo Nordisk, Princeton, NJ, USA) from one up to three injections (adding one injection every 16 weeks) in patients with T2D failing OAMs with or without basal insulin, but this study did not have a comparator. By evaluating the addition of ≥1 mealtime insulin injection, employing randomization and active control and involving many countries, the findings in this study may have broader generalizability.

A limitation of the present study is the open-label design, which was unavoidable given the cloudy vs. clear appearance of the insulin preparations that prevented blinding. This may have contributed to inequality in the numbers of injections given that most practitioners use premix insulin twice daily and insulin glargine once daily. While the protocol allowed titration up to three to four injections, most patients only went up to two injections in spite of endpoint A1Cs not being <7%. It could be argued that tighter control should have been implemented considering that lower A1Cs were achievable with three to four insulin injections, as shown in Rosenstock et al. [15]. The protocol included an option to switch LM50/50 to LM75/25 if FBG targets were not achievable, and some patients who switched achieved lower A1Cs. More stringent guidelines in the study design for switching to LM75/25 may have been warranted.

The slight worsening of glycaemic control in the G+L group after 24 weeks emphasizes the importance of being vigilant about getting patients to achieve glycaemic control. The wide variability in the change in A1C (95% CI: −0.03, 0.37) reflects the broad differences in patients' responses to these insulin regimens. Other factors may need to be studied to better understand this. The apparent resistance to increasing numbers of injections is a reminder that there are barriers to intensifying insulin therapy that also may need to be considered and addressed to more effectively aim for better glycaemic control earlier in the natural history of T2D. One of these barriers is clinical inertia or failure of health-care providers to initiate or advance therapy in a patient who is not at the recommended therapeutic goal [19,20].

In summary, this study shows that both the PP and G+L regimens are efficient in lowering A1C even if non-inferiority of PP to G+L could not be shown. These findings support the belief that most patients with T2D will likely need more than one type of insulin [5,10], and that targeting both fasting and mealtime BG levels is important. These findings may aid physicians as they choose and optimize insulin regimens for individual patients with T2D.
